# New Diabetic Medication Sodium-Glucose Cotransporter-2 Inhibitors Can Induce Euglycemic Ketoacidosis and Mimic Surgical Diseases: A Case Report and Review of Literature

**DOI:** 10.3389/fsurg.2022.828649

**Published:** 2022-03-24

**Authors:** Antonia-Therese Kietaibl, Peter Fasching, Karl Glaser, Alexander H. Petter-Puchner

**Affiliations:** ^1^Department of 5th Internal Medicine With Endocrinology, Rheumatology and Geronotology With Outpatient Department, Clinic Ottakring, Vienna, Austria; ^2^Department of General-, Oncologic- and Visceral Surgery, Clinic Ottakring, Vienna, Austria

**Keywords:** diabetes mellitus, diabetic ketoacidosis, surgery, perioperative, sodium-glucose cotransoporter-2 inhibitors

## Abstract

**Background:**

Euglycemic diabetic ketoacidosis (EDKA) is a potentially life-threatening condition and a reported side effect of antidiabetic sodium-glucose-cotransporter-2-inhibitors (SGLT2-I). The analysis of the herein presented case and its management formed the incentive to prepare this multidisciplinary work and includes an overview about perioperative SGLT2-I-induced ketoacidosis.

**Method:**

A PubMed search on relevant entries was conducted combining the terms “euglycemic diabetic ketoacidosis” AND “surgery.”

**Results:**

A total of 33 articles on SGLT2-I-induced ketoacidosis in the context of surgical treatment were identified. According to this literature research risk factors for the development are infection, perioperative fasting, surgical stress, and insulin dose reduction.

**Conclusion:**

Unspecific symptoms mimicking acute abdomen and normoglycemia can lead to delayed diagnosis of EDKA and might harm patients under SGLT2-I therapy in the perioperative setting. SGLT2-I medication should be withheld for at least 24–48 h prior to surgery according to this review of literature and restarted only in stable clinical conditions to avoid the severe complication of EDKA.

## Introduction

Diabetic ketoacidosis (DKA) is a potentially life-threatening condition and sometimes hard to identify when other seemingly related parameters, such as hyperglycemia, are missing. Furthermore, a correct diagnosis can be difficult and delayed when ketoacidosis presents with unspecific symptoms, such as the picture of an “acute abdomen” (1, 2). The condition of acute abdomen describes many complex symptoms, usually dominated by severe pain and elevated tension (defense) of the abdominal wall. Related or unrelated phenomena include nausea, vomiting, weakness, tachypnea, and tachycardia (3). These clinical features are potentially triggered by DKA and can lead to the suspicion of surgical diseases. As a result, affected patients might be admitted to surgical departments where they undergo operations without any intraoperative correlate (4, 5).

Diabetes mellitus (DM) itself is a well-established risk factor and/or comorbidity for several pathologies seen in surgical wards, such as gastrointestinal malignancy, abdominal, and/or peripheral artery disease, likely with amputation and obesity with bariatric surgery (6–8).

Sodium-glucose cotransporter-2 inhibitors (SGLT2-I) are a modern drug class within the horizon of antidiabetic management. They have been approved by the European medicines agency (EMA) and food and drug administration (FDA) between 2012 and 2014 for the treatment of diabetes mellitus type 2 (T2DM) (9–12). The so-called “gliflozines,” such as ertugliflozin, empagliflozin, dapagliflozin, and canagliflozin, have demonstrated beneficial effects besides their glucose- and hemoglobin A1c (HbA1c) lowering impact. Cardiovascular outcomes studies determined cardio-renal benefits in patients with and without DM (13–18). SGLT2-I use is expected to increase as a consequence of the modifications to present guidelines and a rise in the incidence of global diabetes burden (19–21). Hence, surgeons and anesthesiologists should also be aware of the possible risks and side effects of this drug class. Besides urinary tract and genital infections, one major adverse effect is the so-called euglycemic diabetic ketoacidosis (EDKA), which requires close attention (22–24). Diagnosis of EDKA might be challenging due to the normoglycemic to slightly elevated blood sugar values (<250 mg/dl) and various other potential triggers causing metabolic acidosis (25). When it comes to pathogenesis, EDKA is triggered by relative or absolute insulin deficiency and subsequently increased ketogenesis induced by SGLT2-inhibition, leading to metabolic acidosis (26). Potential triggers include infection, malignancy, diet change, fasting, and perioperative stress (27–29). At our hospital, a patient suffering from both acute appendicitis and newly-diagnosed diabetes mellitus with a blurred DKA due to SGLT2-I initiation and in a serious condition was treated at the surgical department. The analysis of the case and its management formed the incentive to prepare this multidisciplinary article and give a brief overview of available literature about SGLT2-I induced EDKA in the surgical and perioperative context.

### Case Presentation

#### Patient Demographics, Chief Complaint, History, and Diagnostic Assessment

A 39-year-old man [body mass index (BMI) 26.58 kg/m^2^] was admitted to the surgical outpatient ward with abdominalgia, mostly affecting the lower right abdomen (visual analog scale (VAS) 7/10) since the previous day. At presentation, the patient was afebrile (36.7°C) and vital parameters were unremarkable. The cardiopulmonary examination was non-contributory, but palpation was painful in the lower abdomen and McBurney's sign was positive. Defecation and micturition were normal and the patient denied nausea or emesis. He did not suffer from any prior chronic diseases and did not take any long-term medication; no allergies were known but nicotine abuse (20 cigarettes per day) was reported. After physical examination, lab tests of blood and urine and a diagnostic ultrasound were performed to validate the suspected diagnosis of acute appendicitis.

#### Results, Interventions, and Follow-Up

Blood workup revealed elevated infection parameters (C-reactive protein (CRP) 24.2 mg/L (0.0–0.5 mg/L), white blood cell count (WBC) 19.9 G/L (4.00–10.00 G/L), and elevated random glucose 272 mg/dl (60–110 mg/dL), but a normal hemogram, kidney function, electrolyte profile, and liver parameters (Table 1). Testing for severe acute respiratory syndrome coronavirus type 2 (SARS-CoV-2) was negative throughout the whole inpatient stay. Abdominal ultrasound confirmed the diagnosis of acute appendicitis and the patient was admitted to the surgery ward. He was prescribed antibiotic therapy [amoxicillin/clavulanic acid 2.2 grams intravenous (iv)] and was transferred to the operation theater for appendectomy under general anesthesia on the admission day. The laparoscopic approach had to be converted to a lower median laparotomy due to secondary peritonitis as a result of perforated appendicitis [Mannheim peritonitis index (MPI) 12 points (30)]. After surgery, the patient was readmitted to the ward and received analgetic therapy (acetaminophen plus metamizole on request up to three times a day), antithrombotic prophylaxis (low molecular weight heparin once daily), and IV (antibiotic therapy was continued). Histologic results confirmed gangrenous appendicitis with periappendicitis, perforation, and fibrinous-putrid peritonitis. Microbiological analysis of intraabdominal swabs showed growth of *Escherichia coli*.

**Table 1:** Laboratory parameters of interest in course of time.

**Laboratory findings**										
**Date**	**CRP**	**WBC**	**BG**	**pH**	**sO2**	**Lactate**	**HCO3–**	**K+**	**BE**	**AG**
Admission Day	24.2	19.00	272	–	–	–	–	–	–	–
Day 2	180.0	22.74	205	–	–	–	–	–	–	–
Day 3	294.0	15.69	124	–	–	–	–	–	–	–
Day 4	–	–	173	7.13	99.8	0.9	8	4.3	−19.5	19.0
Day 5	161.0	11.08	140	7.36	96.7	0.5	17.6	3.9	−10.0	7.0
Day 6	128.0	11.28	146	7.405	95.9	0.5	19.4	3.7	−7.7	10.0
Day 7	–	–	–	7.461	94.8	0.6	25.7	3.7	1.2	9.0
Day 8	52.1	11.00	129	7.471	95.3	0.8	23.5	3.9	−2.2	11.0
Day 11	15.1	10.48	112	–	–	–	–	–	–	–

*CRP, C-reactive protein (0.0–0.5 mg/l); WBC, white blood cell count (4.00–10.00 G/l); BG, blood glucose (60–110 mg/dl); pH (7.35–7.45); sO2, oxygen saturation (94–98%); Lactate (<1.7 mmol/l); HCO3-, bicarbonate (21–28 mmol/l); K+, potassium (3.4–4.5 mmol/l); BE, base excess (−2–3 mmol/l); AG, Anion gap [Na−(Cl+HCO3), 3–11 mmol/l]*.

Additional lab results showed HbA1c of 12.8% (4.0–6.0%) and vitamin-d deficiency. Therefore, the patient was seen by the consulting physician, and a combination of one thousand/5 mg metformin/empagliflozin and vitamin d supplements for vitamin D deficiency were prescribed and started. On postoperative day (POD) 2, the patient presented with respiratory deterioration (oxygen saturation 93%, tachypnea, still negative for SARS-CoV2). Chest X-ray revealed left-sided pneumonia. Antibiotic treatment was continued and oxygen therapy started (nasal cannula up to 3 l per minute). On POD 3, the patient presented with vomiting, nausea, and abdominal defense and secretion from the intraabdominal drain. The decision to revise the operating field was made. However, no remarkable findings were detected intraoperatively, the repeated microbiological workup confirmed the growth of *E. coli* (explainable by the residual situation after perforated appendicitis). In the recovery room, the patient was first diagnosed with metabolic acidosis (Table 1) and changed breathing patterns with rest dyspnea, abdominalgia, and polyuria. The urine dipstick showed positive results for ketones, protein, and glucose, and the repeated blood gas analysis (BGA) confirmed high anion-gap acidosis (Table 1). Hence, therapy for metabolic acidosis was started in accordance with the department for endocrinology, including IV fluids (IVFs), IV continuous insulin (0.5 ml per hour with short-acting insulin), potassium substitution, and 5%-glucose solution. SGLT2-I therapy was discontinued subsequently on POD 4 after two doses of empagliflozin, and BGA showed slow recompensating of the metabolic state within the next 24 h (Table 1). On POD 4 the patient was retransferred to the surgical ward, balanced BGA was documented on POD 6 and IVFs (IV insulin, glucose, and potassium) were stopped consequently. Infection parameters were declining and pain management was sufficient. Diabetes management was changed. We discontinued empagliflozin/metformin and started linagliptin/metformin 2.5 mg/one thousand mg after abdominal ultrasound (for exclusion of pancreatic pathologies). The patient received diabetes education and testing for autoimmune diabetes (antibodies to glutamic acid decarboxylase (anti-GAD), islet cell antibodies (anti-ICA), and tyrosine phosphatase; C-peptide and insulin) revealed no remarkable results. On POD 11, the patient was discharged. A follow-up appointment at the outpatient department of abdominal surgery showed a clean wound situation. The patient was instructed to visit the outpatient department of endocrinology 3 months after hospital discharge for further discussion of results and management; however, he did not show up to the follow-up appointment.

## Methods: Literature Research

The medical literature search was conducted in PubMed to determine published articles for SGLT2-I-induced EDKA in the surgical setting. These terms were combined: “euglycemic diabetic ketoacidosis” AND “surgery.” There were no restrictions concerning publication date (until January, 2022), article type, or study design. The following inclusion criteria were applied: availability of free full texts in the English or German language. Hence, exclusion criteria consisted of abstracts only and articles published in other foreign languages and the non-use of SGLT2-I or lack of perioperative context.

## Results

### Literature Research

**Figure 1:**
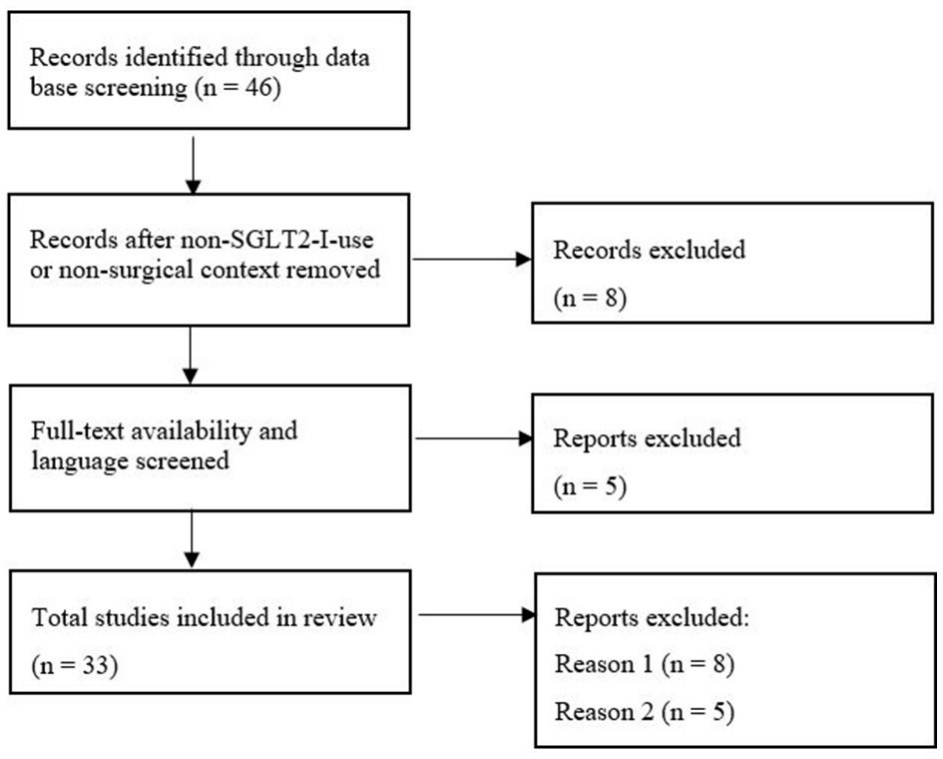
Flowchart of process of literature search.

PRISMA flowchart (Figure 1) shows the initial 46 results from which 13 were excluded due to the missing use of SGLT2-I or surgical/perioperative context and unavailability of free full text or English translation. In total 33 publications (17 case reports, 7 case series, and 9 reviews) were identified. Supplementary Table 1 sums up the literature and important key points and highlights recommendations for perioperative use.

### Characteristics of Patients and Identified Surgeries

The identified papers included reports about bariatric (1, 27, 31–37), cardiothoracic (2, 38–46), neuro- (47–49), vascular (50), orthopedic (51), urologic (52), ophthalmologic (53), and visceral (2, 49, 54, 55) surgery. Most of the interventions were elective, and therefore the time of withhold and restart were predominantly mentioned. The onset of EDKA was recognized between POD 0 [withhold of SGLT2-I for 48 h prior to surgery (38)] and 6 weeks after [under SGLT2-I intake (1)] surgery, hence, reflecting a wide range of clinical presentation. Reported patients were between 42 (31) and 76 (2) years of age, were prescribed empagliflozin (2, 33, 37–45, 47–50, 55), ertugliflozin (53), canagliflozin (2, 31–33, 35, 36, 49, 54), or dapagliflozin (1, 27, 35, 41, 46, 51, 54), and female gender was an assumed risk factor (56). A long history of diabetes duration (>10 years) and the use of oral antidiabetics with or without insulin application were reported as predisposing patients' characteristics (57). Regarding common symptoms, tachypnea, tachycardia, nausea, vomiting, general weakness, and abdominal pain were described; however, specific symptoms were lacking in EDKA (58).

### Pathophysiology and Risk Factors

Sodium-glucose-cotransporter-2-inhibitors lead to glucosuria by inhibiting the sodium-glucose-cotransporter-2 in the renal proximal tubule. As a result, ketone reabsorption increases (17, 26). Pathogenesis of EDKA is triggered by relative or absolute insulin deficiency and subsequently increased ketogenesis, leading to metabolic acidosis. As iatrogenic-induced glucosuria is the consequence of SGLT2-inhibition, blood glucose levels may remain near to normoglycemic in EDKA (26, 59). With a so-called second hit trigger that promotes a ketogenic metabolic state, ketoacidosis can be achieved in times of relative/absolute insulin deficiency (25, 41). Thus, some precipitating factors have been identified in the literature (60). In patients with T2DM on SGLT2-I therapy, ketone levels might reach dangerous levels once stress, infection, or perioperative fasting are present (25). Reported risk factors are prolonged fasting (27) and/or reduced oral intake (33) due to vomiting or dietary changes prior to weight loss surgery (very-low-calory diet) (34), huge weight loss after bariatric surgery (27), acute infection (38), basal insulin discontinuation and/or dose reduction before and/or after surgery (27, 31, 32), continuation of SGLT2-I medication perioperatively (46, 55), or withholding within 24–48 h prior to elective interventions (47, 54). Patients undergoing bariatric surgery might be at special risk for the development of EDKA because of surgical stress, perioperative diet changes, and prolonged fasting periods (31, 34, 36, 37, 52, 59, 61). The incidence of EDKA in diabetic cardiac bypass surgery patients indicates a worrisome potential of this side effect (2, 38–46).

### Perioperative Recommendations & Diagnostic Assessment

The use of SGLT2-I during periods of fasting, illness and major surgery, or low-carb diets should be avoided (27). It might be important to obtain serum and urine ketones in patients with SGLT2-I use and nausea and/or vomiting to early detect cases of EDKA (1) and measure urine ketones once gastrointestinal symptoms appear (47). In major elective or emergency surgery, the early onset infusion of insulin and glucose instead of subcutaneous insulin sliding scales could help prevent EDKA (42, 44). Patients' education to stop SGLT2-I 48 or rather 72 h prior to any surgery might help prevent EDKA (40, 47, 59). In fact, there is a pharmacological rationale for withholding SGLT2-I longer than the commonly recommended 24–48 h due to a longer washout time (31). The most important parameters to diagnose EDKA are relative euglycemia (<250 mg/dl), acidosis (pH <7.30, bicarbonate <18 mEq/l), and the assessment of ketosis. Resolution is characterized by pH > 7.30, normal food intake, closed anion gap (<12 mmol/l), and serum bicarbonate within normal ranges (61). Post-operatively, restart of SGLT2-I after EDKA resolution should only occur with caution on a patient-based basis. History of SGLT2-I-induced EDKA must be added to the patient's allergy chart (36, 61). Patients need to be aware of the potential side effects in predisposing situations and be able to follow “sick day rules” (36, 56, 59).

## Discussion

In this work, we present the case of a patient with acute appendicitis, peritonitis, and a newly-diagnosed T2DM. Persisting abdominal pain led to a second-look surgery which yielded no intraabdominal abnormalities. The authors suggest that postoperative abdominal pain in this patient was the side effect of two doses of empagliflozin, which has been established in the perioperative course. The patient was diagnosed with EDKA on POD3 and might have developed acidosis earlier with delayed recognition due to normoglycemia. In the following discussion, we want to emphasize the possible pitfalls of SGLT2-I use in the surgical context.

Diabetes is more often treated by SGLT2-I due to the results of recent cardiovascular outcome studies demonstrating the positive effects on renal outcomes and improvement in hospitalized heart failure as well as decreasing cardiovascular mortality and overall mortality in patients with or without DM. Following this, SGLT2-I importance and clinical confrontation are obvious (13–16). The presented patient was prescribed with a combination therapy of metformin and empagliflozin due to newly-diagnosed T2DM, which is in accordance with recent guidelines (20).

This work shall raise the awareness that besides the proven beneficial effects of SGLT2-I (such as lowering of blood glucose, systolic blood pressure, and body weight), serious side effects can occur: iatrogenic-induced glucosuria might lead to genital fungal and/or urinary tract infections (22), and rare cases of Fournier's gangrene have been reported (23, 52). On top, reports about SGLT2-I-induced EDKA are rising (54, 62). Diabetic ketoacidosis is known to cause diffuse abdominal pain and may mimic the picture of acute abdomen, which has already been described by Campbell et al. (5). Munro et al. first pictured the condition of DKA without hyperglycemia in 1973 (63). There is growing literature about EDKA in surgical patients receiving SGLT2-I treatment (58, 61). Onset and diagnosis of EDKA can differ due to a wide range of unspecific clinical symptoms explaining its diagnostic challenge (25). In our patient, it took several days to identify the metabolic acidosis because the respiratory deterioration was linked to X-ray confirmed pneumonia. The patient was euglycemic and the symptom of abdominal pain was interpreted as a post-operative reaction. Thus, the persisting abdominal pain aggravated to mimicry of the acute abdomen and led to a second-look laparotomy prior to diagnosis of EDKA. Unspecific symptoms, such as abdominal pain and tachypnea, with euglycemic state, make the diagnosis of EDKA challenging (34, 54). Goto et al. reported two cases of delayed diagnosis, leading to life-threatening complications following EDKA (2).

In a retrospective analysis of our case, we think that the performance of BGA at the time of onset of abdominal pain and worsening respiratory state might have revealed metabolic acidosis earlier and second-look surgery could have been avoided. Although the patient had HbA1c >12% and newly-diagnosed DM in the perioperative context with acute infection, SGLT2-I therapy was started and continued for 2 days. Hence, quite a few risk factors have been presented in this patient that contributed to the development of SGLT2-I-induced EDKA. The patient suffered from appendicitis, peritonitis, and postoperative pneumonia. Acute infection is associated with an increased endocrine stress reaction, thus promoting insulin deficiency with expanded risk for EDKA (25). Kithara et al. reported the first case of intraoperatively diagnosed EDKA with bacterial empyema and fever (38). In this case, perioperative fasting and surgical disease were predisposing factors. In the perioperative period decreased oral intake and/or fasting and surgical stress contributed to a ketogenic state and glucagon secretion with consequent insulin resistance (41).

One must not forget that SGLT2-I-induced ketogenesis also occurs in the absence of the surgical context and can even be therapeutically advantageous (64). Increased ketone body circulation due to a shift to ketone metabolism in SLGT-2 use is one of the several potential hypotheses explaining the cardio-renal protective benefits (65, 66). EDKA; As adverse effect, was very rare in cardiovascular outcome studies under study conditions (67, 68). Osafehinti et al. suggest to anticipate stress levels of undergone procedure (40). Pace et al. report two cases undergoing pancreatectomy with EDKA despite withholding of SGLT2-I 24 h before surgery (54). Herein, appendectomy was enough to develop SGLT2-I-induced EDKA. The authors want to point out that two doses of empagliflozin in combination with the above-mentioned risk factors have been enough to trigger EDKA. After second-look surgery, the patient was readmitted to the ward with suspicion of late-autoimmune diabetes, without turning attention to the potential impact of SGLT2-I use. It would have been helpful to be aware of the possibility of SGLT2-I-induced EDKA.

The symptom of abdominal pain in the postoperative context might cause non-conclusive diagnostic imaging or inadequate surgery (31, 32). In the presented case, relaparotomy was indicated due to the imitation of the acute abdomen also known as “pseudoperitonitis diabetica” (4). Iqbal et al. published a case series of SGLT2-I-induced EDKA in three patients after bariatric surgery leading to computer tomography and/or angiography, whereas Lane et al. reported a case with explorative laparoscopy due to unclear postoperative abdominalgia (31, 34). None of these procedures were contributory and would have been avoidable with earlier recognition and diagnosis of EDKA. To minimize the potential risk of EDKA and delay in diagnosis, a possible solution might be to assess ketones routinely on admission day and postoperatively under the present use of SGLT2-I (69).

The herein presented patient was slightly overweight and had new-onset diabetes. DM is one of the major comorbidities indicating weight loss surgery. Global diabetes burden is increasing as is worldwide SGLT2-I use. Therefore, the perioperative management of SGLT2-I and EDKA awareness in this field needs to be strengthened (8, 21, 34). In their narrative review, Long et al. reported 20% of postoperative EDKA cases following bariatric surgery in insulin-dependent diabetes (61). In the future, earlier bariatric surgery in severe obesity is to be expected. Confrontation of obesity and diabetes (so-called “diabesity”) and perioperative SGLT2-I use might increase accordingly (8, 19). Hence, Iqbal et al. demanded specific guidelines for the management of SGLT2-I in bariatric surgery (34). Lane et al. concluded that patients undergoing bariatric surgery might be at special risk for the development of EDKA because of surgical stress, perioperative diet changes, and prolonged fasting periods (31).

Lack of evidence regarding recommendations for perioperative management of SGLT2-I leads to an eminence-based approach. According to the Austrian Diabetes Society (ÖDG) metformin and SGLT2-I should be paused 24 h before planned surgery and 48 h prior to a major operation. The ÖDG suggests to restart oral antidiabetic medication once the acute situation has stabilized, infections are healed, or earliest on POD 1. (70) In contrast, the American Diabetes Association (ADA) recommends to withhold SGLT2-I 3–4 days prior to operation, and Pace et al. suggest to discontinue 4 days before operation due to a long half-life period (54, 71). The American College of Endocrinology (ACE) and the American Association of Clinical Endocrinology (AACE) advocate to withhold SGLT2-I 24 h before surgery, whereas the Australian and New Zealand College of Anesthetists and Australian Diabetes Society suggest discontinuation 3 days before elective surgery (72–74). The FDA updated safety labels in 2020 and goes in line with the recommendation to withhold 3 days prior to surgery (62). Milder et al. emphasize cessation in case of bariatric surgeries with preoperative dietary changes and recommend to discontinue 1–2 weeks prior to surgery (75). Taking into account the half-life time of 8–16 h of SGLT2-I and the need for corporal elimination of about five half-lives, withholding 24–72 h might not be long enough to avoid EDKA (25, 41). Therefore, the need for further studies to address this important and potential life-threatening complication of SGLT2-I use is obvious. The reported incidence of DKA secondary to SGLT2-I use is 0.1–0.3%, increasing to 19–28% in the perioperative context due to caloric restriction and surgical stress with subsequent ketogenesis (2, 51, 76). Thus, future guidelines should raise awareness and revise the specific management of SGLT2-I in the surgical setting.

## Conclusion

Taking into account the potential risk factors and pharmacologic rationale of SGLT2-I-induced EDKA, we recommend to closely monitor BGA and be especially aware of the side effect. It might be beneficial to assess BGA in patients with DM and those who routinely use SGLT2-I in the preoperative setting to determine the individual ketogenic state. In our patient, diagnosis of EDKA was delayed due to the euglycemic state, abdominal pain in the postoperative setting, mimicry of acute abdomen in the context of peritonitis, and late assessment of BGA. Again, we want to emphasize the symptom of “diabetic pseudoperitonitis” in patients with ketoacidosis, which leads to a second-look laparotomy because of mimicry of the acute abdomen on POD 3 in the present case. To conclude, we present the case of a patient with acute appendicitis, peritonitis, and new-onset diabetes diagnosed with SGLT2-I-induced EDKA postoperatively. Our case belongs to the spectrum of moderate EDKA with delayed diagnosis due to unspecific symptoms, mimicry of acute abdomen, and relatively normoglycemia. In the perioperative setting, SGLT2-I therapy should be withheld for at least 24–48 h and restarted with caution to avoid this severe complication according to the guidance of the national diabetes society. However, it might be reasonable to hold SGLT2-I more than 48 h before surgery taking into account pharmacokinetics and the above-mentioned perioperative risk factors.

## Data Availability Statement

The original contributions presented in the study are included in the article/Supplementary Material, further inquiries can be directed to the corresponding author/s.

## Ethics Statement

Due to the retrospective character of this case report, and the fact that every patient signs an informed-written consent form at the time of admission, written informed consent was not needed from the individual(s) for the publication of any potentially identifiable images or data included in this article.

## Author Contributions

The manuscript was written by A-TK and AP-P and reviewed and edited by PF and KG. A-TK, PF, KG, and AP-P contributed to the design and implementation of the research. All authors have seen and approved the submission of this version of the manuscript and take full responsibility for the manuscript.

## Conflict of Interest

The authors declare that the research was conducted in the absence of any commercial or financial relationships that could be construed as a potential conflict of interest.

## Publisher's Note

All claims expressed in this article are solely those of the authors and do not necessarily represent those of their affiliated organizations, or those of the publisher, the editors and the reviewers. Any product that may be evaluated in this article, or claim that may be made by its manufacturer, is not guaranteed or endorsed by the publisher.

## Abbreviations

BGA: blood gas analysis
ACE: American College of Endocrinology
AACE: American Association of Clinical Endocrinology
anti-GAD: antibodies to glutamic acid decarboxylase
anti-ICA: antibodies to islet cells
BE: Base excess
BMI: Body mass index
CRP: C-reactive protein
DKA: Diabetic ketoacidosis
DM: Diabetes mellitus
EDKA: Euglycemic diabetic ketoacidosis
EMA: European medicines agency
FDA: Food and drug administration
HbA1c: Hemoglobin A1c
Iv: Intravenous
IVFs: Intravenous fluids
MPI: Mannheim Peritonitis Index
ÖDG: Austrian Diabetes Society
POD: postoperative day
SARS-CoV-2: severe acute respiratory syndrome coronavirus type 2
SGLT2-I: Sodium-glucose-cotransporter-2-inhibitors
T1DM: Type 1 diabetes
T2DM: Type 2 diabetes
VAS: visual analog scale
WBC: white blood cell count.
