# Supplementary material for: New Diabetic Medication Sodium-Glucose Cotransporter-2 Inhibitors Can Induce Euglycemic Ketoacidosis and Mimic Surgical Diseases: A Case Report and Review of Literature
Source: Front Surg. 2022 Mar 24;9:828649. doi: 10.3389/fsurg.2022.828649 (PMC8987984; doi:10.3389/fsurg.2022.828649)
Supplement: Supplementary file 1 [file Table_1.pdf]

Supplementary Material

1 Supplementary Material

1.1.1 Supplementary TABLE 1. Literature relating SGLT2-I induced EDKA in perioperative context

**Author & Publication year**	**Patient(s) & symptoms**	**Risk factors**	**Perioperative recommendation**
Case Reports			
Banakh et al., 2019	Pat: 64a, female, bariatric surgery 4w before<br><br>Antidiabetic agents: insulin, metformin, dapagliflozin<br><br>Lab/symptoms: pH 6.93, HCO3- 2, BG 243, shortness of breath, lethargy, vomiting, reduced oral intake, dehydration, tachycardia	- prolonged fasting<br><br>- large weight loss after surgery<br><br>- surgery	- avoid SGLT2-I use in T1D, during prolonged fasting, dehydration or illness, major surgery, excess alcohol intake or low carb diets, reducing insulin doses too fast
Brown et al., 2018	Pat: 53a, male, bariatric surgery 6w before<br><br>Antidiabetic agents: metformin,dapagliflozin<br><br>Lab/symptoms: pH 7.24, anion gap 30, BG 162, beta-hydroxybutyrate 6.2, nausea, vomiting, anorexia, weakness, fatigue, generalized abdominal pain, tachycardia	- T2D<br><br>- surgery	- importance of obtaining serum and urine ketones in patient with SGLT2-I and nausea and vomiting
Bteich et al., 2019	Pat: 58a, female, neurosurgery<br><br>Antidiabetic agents: insulin, glipizide, metformin, empagliflozin<br><br>Lab/symptoms: GCS 6, worsened clinical status, metabolic acidosis with high anion gap and serum hydroxybutyrate, pH 7.2, BG 130-150, ketone- and glucosuria	- no enteral nutrition<br><br>- surgery<br><br>- last dose of SGLT2-I 24h before surgery	- cessation of antidiabetic drugs > start long-acting insulin to mitigate the EDKA<br><br>- educate patients to stop 48h prior to surgery, prompt reporting of gastroenteric symptoms and use urine ketone strips to screen
Cha et al., 2021	Pat: 55a, male, prediabetes, orthotopic heart transplant<br><br>Antidiabetic agents: dapagliflozin<br><br>Lab/symptoms: pH 7.26, HCO3- 16, BG 173, AG 28,	- SGLT2-I discontinued after surgery<br><br>- high dose steroids, fasting	- EDKA in prediabetic patient is possible<br><br>- some surgical procedures are not elective > new challenges in the perioperative risk profile

		- cardiac surgery	
Chandrakumar et al., 2021	Pat: 68a, female, ophthalmologic surgery<br><br>Antidiabetic agents: ertugliflozin started right before surgery<br><br>Lab/symptoms: pH 7.36, AG 23, HCO3- 4.89, BG 110, urine ketones 80, decreased appetite, malaise, constipation	- surgery, infection, low oral intake	- delayed diagnosis due to normoglycemia
Gomez-Sanchez et al., 2021	Pat: 73a, male, vascular surgery<br><br>Antidiabetic agents: empagliflozin, insulin metformin<br><br>Lab/symptoms: pH 7.2, HCO3- 8.9, BE -16.7, BG 170-200, glucosuria, ketonuria, acute delirium POD2, hypotensive	- last dose of SGLT2-I 24h before surgery<br><br>- no insulin on POD1	- hold SGLT2-I 48h prior to surgery<br><br>- emphasizing early postoperative oral intake<br><br>- continue basal insulin
Ito et al., 2021	Pat: 55a, male, cardiac emergency surgery<br><br>Antidiabetic agents: empagliflozin, metformin, glimepiride, vildagliptin, acarbose<br><br>Lab/symptoms: pH 7.25, BG 149, high anion gap acidosis, ketonemia, ketonuria	- SGLT2-I not withhold<br><br>- cardiac, emergency surgery	- insulin and glucose infusion could prevent EDKA in case of emergency surgery
Kapila and Topf, 2021	Pat.: 51a, female, bariatric surgery<br><br>Antidiabetic agents: insulin aspart, insulin glargine, metformin, and canagliflozin<br><br>Lab/symptoms: pH 7.21, HCO3- 8, AG 37, BG 150-180, ketonuria, glucosuria, lethargy, tachycardic, hypertensive	- bariatric surgery<br><br>- ketogenic diet three weeks prior to surgery<br><br>- last dose 48h prior to surgery	- SGLT2-I use must be managed carefully in the perioperative period<br><br>- Strengthen awareness and education of predisposing risk and perioperative management<br><br>- Add to patient´s allergy list in case of EDKA after SGLT2-I use
Kitahara et al., 2021	Pat: 59a, male, BMI 24, thoracic surgery<br><br>Antidiabetic agents: empagliflozin, intensive insulin therapy<br><br>Lab/symptoms: pH 7.15, HCO3- 16.7, BG 162, lactate 1.2, glucosuria, ketonuria	- last dose of SGLT2-I 28h, last insulin 13h before surgery<br><br>- fasting + surgery, acute infection	- in case of insufficient withdrawal period of SGLT2-I > monitor ketones + acidosis

Kuchay et al., 2021	<u>Pat:</u> 53a, female, BMI 19, cardiac surgery<br><br><u>Antidiabetic agents:</u> vildagliptin, metformin, empagliflozin<br><br><u>Lab/symptoms:</u> pH 7.01, HCO3- 10, BG 123, altered mental status, ketonuria, metabolic acidosis	- last dose of oral antidiabetic drugs 48h before surgery<br><br>- surgery	- early recognition and treatment of EDKA can prevent morbidity and mortality<br><br>- more research is needed for appropriate time of withdrawing prior to major surgery
Lane et al., 2018	<u>Pat:</u> 42a, female, BMI 40, bariatric surgery<br><br><u>Antidiabetic agents:</u> canagliflozin, sitaglipitin, metformin, insulin glargine<br><br><u>Lab/symptoms:</u> pH 6.77, HCO3- 2.5, BG 180, lactate 18, AG 28; serum ketones 47, POD2: dyspnea, dizziness, tachycardia, hypertension; POD3: thirst, confusion	- last dose 24h before surgery<br><br>- bariatric surgery, preoperative very-low-calorie diet for 2w<br><br>- insulin intake halved	- pharmacologic rational for “washout” time >24h<br><br>- patients with bariatric surgery may be at special risk: surgery + long fasting perioperatively
Mackintosh et al., 2020	<u>Pat:</u> 68a, female, neurosurgery<br><br><u>Antidiabetic agents:</u> empagliflozin<br><br><u>Lab/symptoms:</u> pH 7.2, HCO3- 9, BG 140-160, AG 21, ketonuria, glucosuria, beta-hydroxybutyrate pos.; POD1: lethargic, worsening aphasia, confusion	- last dose on day before surgery<br><br>- neurosurgery<br><br>- decreased oral intake	- cessation earlier than day of surgery or stricter monitoring and/or treatment with increased insulin doses
Osafehinti et al., 2020	<u>Pat:</u> 60a, male, cardiac surgery<br><br><u>Antidiabetic agents:</u> empagliflozin, glimepiride, metformin, semaglutide<br><br><u>Lab/symptoms:</u> few hours after surgery pH 7.27, HCO3- 15, BG 138, beta-hydroxy 6.52, high AG, preoperative glucosuria and ketonuria	- last dose 48h prior to surgery<br><br>- cardiac surgery	- persistent glycosuria despite SGLT2-I withhold: warrants close monitoring<br><br>- consider discontinuing SGLT2-I at least 48 to 72h before major surgery
Smith et al., 2021	<u>Pat:</u> 51a, female, bariatric surgery<br><br><u>Antidiabetic agents:</u> canagliflozin, metformin, basal-bolus insulin<br><br><u>Lab/symptoms:</u> pH 7.21, HCO3- 3, BG 160, AG 37, ketonuria, glycosuria, serum ketones 49.8; POD1: tachycardia, POD2: altered mental status, tachypnea	- last dose 48h prior to surgery<br><br>- bariatric surgery with preoperative diet change<br><br>- basal insulin discontinuation	- educate patients about signs and symptoms of EDKA<br><br>- studies needed to determine best perioperative management

Wohlrab et al, 2021	Pat: 62a, male, coronary artery surgery<br><br>Antidiabetic agents: empagliflozin<br><br>Lab/symptoms: pH 7.1, BE -14.3, high AG, BG 148, ketonuria, glucosuria, nausea, abdominal pain, hyperventilation	- cardiac surgery<br><br>- last dose of SGLT2-I 24h before surgery<br><br>- fasting	- insulin for intraoperative treatment of hyperglycemia in cardiopulmonary bypasss may delay diagnosis of EDKA<br><br>- prolonged ICU stay due to EDKA<br><br>- need for education and consensus guidance on perioperative use of SGLT2-I
Wong et al., 2021	Pat: 57a, female, orthopedic surgery<br><br>Antidiabetic agents: dapagliflozin, metformin<br><br>Lab/symptoms: pH 6.974, HCO3- 4.2, BG 187, lactate 1.1, POD2: dyspnea, thirst, tachycardia, tachypnea	- last dose on day of procedure<br><br>- restart on POD1<br><br>- orthopedic surgery	- elective surgery: withhold at least 3d; emergency surgery: screen for ketonuria and ketosis<br><br>- restart SGLT2-I once normal diet is possible
Yared and Mancini, 2020	Pat: 49a, male, bariatric surgery<br><br>Antidiabetic agents: empagliflozin, semaglutide, liraglutide, insulin glargine, metformin<br><br>Lab/symptoms: pH 7.106, HCO3- 17.6, BE -24, BG 180, lightheadedness, weakness, slurred speech, tachypnea, tachycardia	- bariatric surgery<br><br>- very low-calorie liquid diet for 2w	- in case of very low-calorie diet EDKA can also occur before bariatric surgery<br><br>- may be appropriate to stop SGLT2-I 2w prior to bariatric surgery
Case Series			
Amianda et al., 2021	Pat: 47a, female, BMI 40, bariatric surgery / 54a, male, BMI 38, bariatric surgery<br><br>Antidiabetic agents: canagliflozin / metformin, semaglutide, empagliflozin<br><br>Lab/symptoms: pH 7.119, HCO3 5.2, BE 21.9, AG 30, ketonuria, normal glucose; shortness of breath, abdominal distention, constipation back pain, tenderness left upper quadrant, tachycardia / pH 7.178, HCO3 6.1, BE 19.9, AG 32, generalized weakness, progressively worsening shortness of breath, dyspnea	- last dose of SGLT2-I on day prior to surgery<br><br>- bariatric surgery<br><br>- decreased oral intake<br><br>- diet change	- bariatric surgery guidelines<br><br>should be updated to ensure clearance of medication to prevent EDKA
Goto et al., 2021	Pat: 52a, female, 51kg, cardiac surgery / 76a, female, 63kg, abdominal surgery	- last dose of SGLT2-I on day before surgery	- potential life-threatening complications caused or worsened by SGLT2i-associated EDKA

	<u>Antidiabetic agents:</u> empagliflozin, sitagliptin / canagliflozin, metformin<br><br><u>Lab/symptoms:</u> pH 6.84, HCO3- 2.1, BG 178mg/dl, BE -20, AG 31.9, lactate 2.4, tachypnea, vomiting, decreased blood pressure, high anion gap / pH 7.25, HCO3- 17.3, BG 100, BE -9, AG 16.2, glycosuria	- low oral intake<br><br>-cardiac /abdominal surgery	- clinicians should pay attention to the discontinuation period of SGLT2i prior to surgical intervention
Iqbal et al., 2020<br><br>- focus on bariatric surgery	<u>Pat:</u> 56a, male, bariatric surgery / 59a, female, bariatric surgery 5w prior/ 52a, female, bariatric surgery 2w prior<br><br><u>Antidiabetic agents:</u> canagliflozin, metformin/ dapagliflozin/ canagliflozin, metformin<br><br><u>Lab/symptoms:</u> pH 6.91, HCO3- 4, BG 208, AG 32, beta-hydroxybutyrate 4.5, generalized, constant, deep aching, moderate abdominal pain, weakness, malaise, polydipsia, polyuria, shortness of breath, tachycardia / pH 7.28, HCO3- 10, BG 173, AG 32, beta-hydroxy 9, lactate 0.8, glucosuria, decreased appetite, nausea, flatus, watery bowl movements / pH 7.20, HCO3- 8, BG 196, AG 35, beta-hydroxy >9, glucosuria, ketonuria, tiredness, shortness of breath, chills, weakness, decreased appetite, chronic polyuria	- bariatric surgery<br><br>- reduced oral intake<br><br>- withhold of insulin<br><br>- diet change	- need for specific guidelines for use after bariatric surgery
Lau et al., 2018	<u>Pat.:</u> 54a, male, coronary artery bypass grafting / 58a, male, coronary artery bypass grafting / 54a, male coronary artery bypass grafting<br><br><u>Antidiabetic agents:</u> NPH insulin, empagliflozin / empagliflozin, metformin, gliclazide / empagliflozin, metformin<br><br><u>Lab/symptoms</u>: pH 7.28, HCO3- 18, BE -8, BG 216, AG 15, nausea, emesis / pH 7.30, HCO3- 17, BE -8, AG 14, BG 112 / pH 7.33, HCO3- 16, anion gap 12, BE -8, BG 173 / all: tachypnea, elevated beta-hydroxybutyrate, glucosuria, ketonuria, tachypnea	- coronary artery bypass surgery<br><br>- fasting<br><br>- reduction in insulin dose<br><br>- EDKA with empagliflozin in 3 cases on POD 1 despite discontinuation 24-48h before surgery	- anesthesiologists need to recognize risk of EDKA<br><br>- discontinuing medication at least two days prior to surgery should minimize the risk<br><br>- urine and serum ketones should be monitored<br><br>- optimize analgetic therapy and volume status to reduce further stress, provide dextrose with insulin to normalize reduced insulin to glucagon ratio<br><br>- Institutional protocols for postoperative ketone monitoring<br><br>- withhold until adequate hydration and normal diet resumes

			- major surgeries: withdraw 3 days
Pace et al., 2018	Pat: 66a, female, abdominal surgery / 75a, male, abdominal surgery<br><br>Antidiabetic agents: canagliflozin, sitagliptin / metformin, glipizide, dapagliflozin<br><br>Lab/symptoms: POD 1: BG 155-224, HCO3- 15, AG 19, beta-hydroxybutyrate 48.1, glucose- and ketonuria, polyuria / BG 158-225, HCO3- 16, AG 19, polyuria, beta-hydroxybutyrate 50.8, glucose- and ketonuria,	- last dose 24h prior to surgery<br><br>-pancreatectomy	- withhold SGLT2-I at least 5 days preoperatively (based upon the long half-life time)
Pontes et al., 2021	Pat: 67a, male, cardiac surgery / 57a, male, cardiac surgery<br><br>Antidiabetic agents: empagliflozin / dapagliflozin<br><br>Lab/symptoms pH 7.25, HCO3- 12.3, BG <200, BE -13.3, high AG 27; POD1: tachypnea, polydipsia, ketonuria, glycosuria, polyuria / pH 7.21, HCO3- 9.4, BG <250, BE -16.6, high AG 30; POD1: tachypnea, unwellness, restlessness, polydipsia, polyuria, ketonuria, glycosuria	- last dose day before surgery<br><br>- cardiac surgery<br><br>- fasting 8h	- 72h suspension of SGLT2-I preoperatively<br><br>- future guidelines: should increase the time of SGLT2-I suspension before major surgery
Rafey et al., 2019	Pat: 44a, male, BMI 38.8, discectomy / 59 a, female, BMI 39, nephrectomy<br><br>Antidiabetic agents: dulaglutide, canagliflozin / insulin glargine, insulin aspart, empagliflozin<br><br>Lab/symptoms**:** pH 7.1, HCO3- 4.8, BG 169, serum ketones 4.3, BG 169, lactate 0.8, AG 33.8, POD1: acidosis, ketonuria, glycosuria, POD6: weakness, lethargy, nausea, anorexia, tachypnoeic, tachycardic / pH 7.23, HCO3- 9.3, BG 222, lactate 0.5, serum ketones 4.8, AG 32.6, POD3: weakness, dyspnea, pre-syncope, tachypnea, tachycardia, ketone- and glycosuria	- surgery<br><br>- did not stop SGLT2-I preoperatively	- surgical patients advised to stop SGLT2-I 48h before procedure and re-start 48h afterwards (the earliest)<br><br>- consider measurement of blood, urine ketones, insulin, c-peptide, glucagon, renal function
Reviews			
Alabdaljabar et al., 2021	Case report + literature review on EDKA and coronary bypass surgery	- last dose of SGLT2-I 24h prior to surgery	- all identified EDKA cases with coronary surgery had SGLT2-I use in common

	Pat: 52a, male, coronary artery bypass surgery<br><br>Antidabetic agents: vildagliptin, metformin, empagliflozin, pioglitazon<br><br>Lab/symptoms: pH 7.22, BG 145-188, HCO3- 14.6, high AG metabolic acidosis, ketonuria	- fasting	- still developed EDKA when withhold SGLT2-I 48h<br><br>- high index of suspicion for EDKA in cardiac patients with SGLT2-I use<br><br>- assess routine urine ketones as screening tool
Bamgboye et al., 2021	2000-2020<br><br>- list of factors predisposing for EDKA<br><br>- existence of variations in SGLT2 receptor expression	- insulin dosage change, alcohol consumption, female gender<br><br>- starvation due to illness or fasting, withhold SGLT2-I less than 48 h	- advise sick day rules for patients<br><br>- local guidelines should be developed for safe perioperative management<br><br>- check urine ketones and plasma for bicarbonate
Chandras-hekar et al, 2021	Review about genitourinary and perioperative implications<br><br>- focus on urological side effects of SGLT2-I: genital infections, urinary tract infections, Fournier´s gangrene, perioperative EDKA	- surgery, fasting, intercurrent illness, medication changes	- SGLT2-I cessation up to 3 days preoperatively<br><br>- consider postponing nonurgent surgery if no SGLT2-I pause, blood ketones >6mmol/l, HbA1c >9.0%<br><br>- EDKA = medical emergency
Goldenberg et al., 2016	01/2013-08/2016<br><br>Reports about DKA with SGLT2 use<br><br>- EDKA rates 0.16-0.76 events per 1000 patient-years in clinical trial programs of SGLT2I;<br><br>- 46 identified case reports<br><br>- Symptoms of EDKA/DKA: thirst, polyuria, nausea, vomiting, abdominal pain, confusion, kussmaul´s breathing, fever, acetone breath	- Insulin reduction, acute illness, dehydration, bariatric or other surgery, low-carb diets,	- SGLT2-DKA: prevention by withholding SGLT2I when precipitants develop<br><br>- bariatric surgery: hold while preop diet<br><br>- major surgery: discontinue 3 days before<br><br>- algorithm for adequate prescription of SGLT2-I
Iqbal et al., 2020	Literature EDKA after bariatric surgery<br><br>Nausea, anorexia, abdominal pain<br><br>- focus on bariatric surgery	- surgery, severe infections, fasting<br><br>- diet, bariatric surgery<br><br>- reduced insulin dosage	- time of onset around 24h<br><br>- literature on post-bariatric surgery cases is growing, time of onset later > 1w<br><br>- no current guidelines regarding antihyperglycemic drugs after bariatric surgery

Long et al., 2021	Narrative review<br><br>- 67 resources included<br><br>Symptoms EDKA: Nausea, vomiting, malaise, fatigue, Kussmaul respirations, dyspnea, fruty odor (breathing), tachycardia, dehydration	- starvation, sepsis, infection,<br><br>- bariatric surgery: 20% of postoperative cases in insulin dependent diabetes	- SGLT2-I restart after resolution of EDKA is possible<br><br>- emergency surgery/nothing by mouth: discontinue
Menghoum et al.,<br><br>*2021*	Review of SGLT2-I EDKA cases 2015-2020<br><br>- 72 included EDKA cases<br><br>- Symptoms: nausea, abdominal pain and<br><br>vomiting	- fasting, surgery<br><br>- acute infection<br><br>- insulin deprivation	- low pH and high b-hydroxybutyrate were associated with EDKA<br><br>- 2.2 times higher risk for EDKA in T2D with SGLT2-I
Nasa et al., 2021	Review about EDKA<br><br>- Symptoms: malaise, tachypnoea, anorexia<br><br>- Diagnosis: pH <7.3, serum bicarbonate <18 mmol/L<br><br>- delayed diagnosis due to normoglycemia	- infection, surgery, perioperative fasting, gastrointestinal surgery, alcohol intake, pregnancy	- withhold 3-4d before planned surgery<br><br>- consider EDKA in unexplained metabolic acidosis in DM and associated risk factors<br><br>- patient education/sick day rules
Sampani et al., 2020	Case report + literature review<br><br>Pat: 51a, female, elective hysterectomy<br><br>Antidiabetic agents: metformin, vildagliptin, empagliflozin<br><br>Lab/symptoms: pH 7.05, HCO3- 3, BG 121, AG 16.9, POD 4-6: vomiting, anorexia, weakness, tachypnea, tachycardia, abdominal pain, POD 6: febrile, polyuria, ketonuria, glycosuria	- fasting POD 1+2<br><br>- no SGLT2-I discontinuation<br><br>- postsurgical cellulitis	- two-hit model for provoking EDKA<br><br>- elective major surgery: withhold at least 3 days prior to surgery<br><br>- administer insulin instead of SGLT2-I perioperatively to prevent EDKA

a, years; AG, anion gap ($Na - (Cl + HCO3$), 3-11mmol/L); BE, base excess (-2 – 3 mmol/L); BG, blood glucose (60-110 mg/dL); d, days; EDKA, euglycemic diabetic ketoacidosis; GCS, Glasgow coma scale; h, hour(s); HCO3-, bicarbonate (21-28 mmol/L); lactate (<1.7mmol/L); pH (7.35-7.45); w, week(s); POD, postoperative day; SGLT2-I, Sodium-glucose-cotransporter-2-inhibitors; T1DM, Type 1 diabetes; T2DM, Type 2 diabetes;
